# Associations between T cells and attention problems in the general pediatric population: The Generation R study

**DOI:** 10.1002/jcv2.12038

**Published:** 2021-10-13

**Authors:** Kirsten I. M. Looman, Charlotte A. M. Cecil, Christina Grosserichter‐Wagener, Jessica C. Kiefte‐de Jong, Menno C. van Zelm, Henriëtte A. Moll

**Affiliations:** ^1^ Generation R Study Group Erasmus MC University Medical Center Rotterdam The Netherlands; ^2^ Department of Pediatrics Sophia Children's Hospital Erasmus MC University Medical Center Rotterdam The Netherlands; ^3^ Department of Child and Adolescent Psychiatry/Psychology Erasmus MC University Medical Center Rotterdam The Netherlands; ^4^ Department of Epidemiology Erasmus MC University Medical Center Rotterdam The Netherlands; ^5^ Molecular Epidemiology Department of Biomedical Data Sciences Leiden University Medical Center Leiden The Netherlands; ^6^ Department of Immunology Erasmus MC University Medical Center Rotterdam The Netherlands; ^7^ Department of Public Health and Primary Care/LUMC Campus The Hague Leiden University Medical Center Leiden The Netherlands; ^8^ Department of Immunology and Pathology Central Clinical School Monash University and Alfred Hospital Melbourne Victoria Australia

**Keywords:** ADHD, attention problems, B cell, immunology, T cell

## Abstract

**Objective:**

The pathogenesis of attention‐deficit/hyperactivity disorder (ADHD) is currently unclear. We hypothesized that chronic immune activation, as indexed by T and B cells, plays a role in the pathophysiology of attention problems. Therefore, we examined T and B cell subsets in a general pediatric population with information on attention problems.

**Methods:**

We included 756 10‐year‐old children from the Generation R population‐based cohort. Eleven‐color flow cytometry was performed on peripheral blood samples to determine T and B cell subsets. The Child Behavior Checklist rated by parents was used to measure attention problems. Data were analyzed using linear regression analyses, adjusting for maternal and child covariates and co‐occurring childhood psychopathology.

**Results:**

For T helper 1 (Th1) cells, one standard deviation (SD) increase was associated with 5.3% (95%CI 0.3; 10.5) higher attention problem scores. Furthermore, 1SD increase in CD8^+^ T cells was associated with 7.5% (95%CI 2.4; 12.7) higher attention problem scores. Within total CD8^+^ T cells, 1SD increase in naive or central memory cells was associated with 6.9% (95%CI 2.0; 12.1) and 6.4% (95%CI 1.5; 11.6) higher attention problem scores, respectively. No associations between Th2, Treg or B memory cells and attention problem scores were observed.

**Conclusion:**

Higher Th1 and cytotoxic T cell numbers are associated with higher attention problem scores independent of co‐occurring psychopathology. This might indicate a possible role of a pro‐inflammatory immune profile in childhood attention problems.

## Graphical abstract

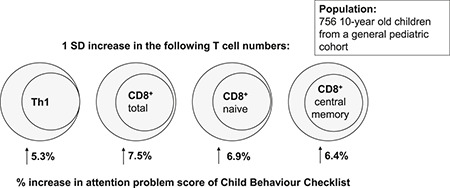

Attention problems have been associated with inflammatory diseases previously, however the exact underlying pathophysiology of ADHD is unclear. This study shows increased T helper 1 and CD8^+^ total, CD8^+^ naive, CD8^+^ central memory numbers in children from a general pediatric population with higher attention problem scores independent of other childhood neurodevelopmental problems.

**Key points**

- Attention problems have been associated with inflammatory diseases previously, however the exact underlying pathophysiology of ADHD is unclear
- This study shows increased T helper 1 and CD8^+^ total, CD8^+^naive, CD8^+^ central memory numbers in children from a general pediatric population with higher attention problem scores
- The observed associations between T cell numbers and attention problems were independent of other childhood neurodevelopmental problems
- These findings might provide a basis for the underlying immunological mechanisms of the association between immune activation and ADHD

## INTRODUCTION

Attention‐deficit/hyperactivity disorder (ADHD) is the most prevalent child behavioral disorder with a prevalence of 5% and an estimated yearly burden of 491,500 disability adjusted life years globally (Erskine et al., 2014; Sayal et al., 2018). ADHD is characterized by hyperactivity, impulsivity and inattention inappropriate for the child's age (Sayal et al., 2018). The clinical diagnosis of ADHD is based on the *Diagnostic and Statistical Manual of Mental Disorders*–5 Task Force criteria in which children should have minimally six of the formulated ADHD criteria before the age of 12 years old (Fairman et al., 2020). The pathophysiology of ADHD is complex and suggestive of an interplay between genetic and environmental factors that impact neurobiological processes (Anand et al., 2017). Growing evidence points to a role of immunological processes as possible additional underlying biological mechanism in the pathophysiology of ADHD (Leffa et al., 2018). Currently, the evidence for the contribution of immunological processes in the pathophysiology of ADHD can be divided in three categories: (1) genetic studies, (2) studies on associations between ADHD and immune‐mediated diseases and (3) cytokine studies (Leffa et al., 2018). Namely, susceptibility genes for autoimmune disease, such as human leukocyte antigen (HLA)‐DR4, HLA‐DRB and complement C4B, have been associated with ADHD (Aureli et al., 2008). Previous meta‐analyses have linked ADHD to both autoimmune and atopic diseases including atopic dermatitis, asthma and allergies (Cortese et al., 2018; Nielsen et al., 2021). Finally, some studies have found that children with attention problems have increased levels of pro‐inflammatory cytokines such as interleukin (IL)‐2, IL‐5, IL‐6, interferon‐gamma (IFN‐ɣ), and tumor necrosis factor (TNF)‐α, suggesting a pro‐inflammatory state (Anand et al., 2017; Cortese et al., 2019; Yu et al., 2020). However, this observation has not been confirmed by all studies (Dozmorov et al., 2018) and in some cases the opposite direction of associations has been identified. For example, Yu et al. (2020) found that higher IL‐8 was positively associated with children's ability to control or regulate attention, emotion, and behavior. Correlations between a pro‐inflammatory state and onset of attention problems form the basis of the hypothesis that there could be a causal relationship between these (Anand et al., 2017). The brain is connected to the peripheral immune system by the lymphatic system (Kipnis, 2016; Morimoto and Nakajima, 2019). Previously, both CD4^+^ helper and CD8^+^ cytotoxic T cells have been shown to be involved in brain development and functioning (Kipnis, 2016). CD4^+^ T cells are thought to contribute to memory development, whereas infiltration of CD8^+^ T cells in the central nervous system disturbs homeostasis in microglial and neuronal activity (Kipnis, 2016; Mohebiany et al., 2020). Such infiltration can occur in a chronic inflammatory state such as in auto‐immune and atopic diseases (Schmidt et al., 2018). CD4^+^ and CD8^+^ T cells belong together with B cells to the adaptive immune system (den Haan et al., 2014). CD4^+^ and CD8^+^ T cells develop from naive to central memory and effector memory cells (den Haan et al., 2014; Sallusto et al., 2004). CD4^+^ memory cells include Th1, which are involved in auto‐immunity, and Th2 and Th17, which are involved in allergic disease (den Haan et al., 2014). B and T cells are closely linked to each other as described in more extent previously (den Haan et al., 2014). Despite the reproducible finding of an association between chronic immune disease and attention problems, insight into the immunological basis is limited. Specifically, immune studies thus far have been restricted to cytokine analyses in small, preselected subsamples and do not include T and B cell numbers in the general pediatric population. Furthermore, previous studies did not account for various confounding factors during the development of the child that might affect the association between immunological mechanisms and attention problems. Finally, co‐occurring psychopathology has not been considered and therefore it is currently unclear whether associations are unique to attention problems or more broadly related to mental health problems. More insight into the potential neuro‐immunological involvement in attention problems could inform future research directions, the understanding of the pathogenesis of attention problems and the development of therapeutic options. Therefore, we here examined the association between B and T cell subsets and attention problem scores dimensionally, based on data from over 700 10‐year‐old children from the general population, with adjustment for confounders and consideration of effect modification by sex.

## MATERIALS AND METHODS

### Design and ethical considerations

**FIGURE 1:**
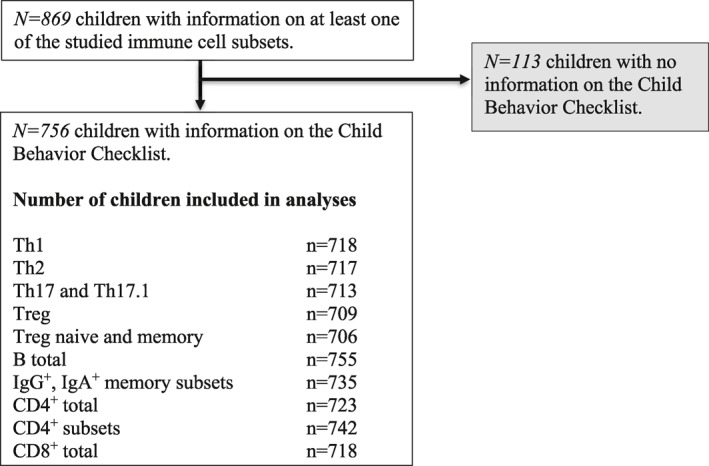
Flowchart of participants included in the study

The study is part of the Generation R Study, a population‐based prospective birth cohort study located in Rotterdam, the Netherlands (Kooijman et al., 2016). The Medical Ethical Committee of the Erasmus University Medical Center in Rotterdam approved the study (MEC‐2012‐165). Written informed consent from legal representatives was obtained from all participants. At child's age of 10 years, data collection was performed in 7393 children. 5862 children visited the research center (response rate 79%), of which 4593 children provided blood samples. In 950 children detailed immune phenotyping was performed. The sample number for detailed phenotyping was restricted as a result of a maximum of 3 samples that could be processed in the laboratory per day. For the CBCL, mothers of 5398 children answered the questionnaire at 10 years (response rate 73%) (Kooijman et al., 2016). For the present study, we selected all children with information on at least one of the measured immune cells and with information on the attention problem score by means of the Child Behavior Checklist (CBCL/6‐18) This resulted in a number of 756 children for the analyses (Figure 1).

### Assessment of immune cells

Peripheral blood samples were obtained at a median age of 9.8 [25%–75% range 9.6; 9.9]. First, a diagnostic lyse‐no‐wash protocol (BD Biosciences, San Jose, CA) was used to obtain absolute counts of peripheral blood B and T cells from peripheral blood samples. Next, 11‐color immunophenotyping was performed on the four‐laser LSR Fortessa (BD Biosciences) (Kalina et al., 2012; Looman et al., 2020). Within the total CD19^+^ B cells, we obtained the following subsets of CD19^+^CD21^+^D38^dim^IgD^−^ memory B cells: CD27^+^IgA^+^ and CD27^−^IgA^+^ (Heeringa et al., 2018). In addition, transitional B cells (CD19^+^CD27^−^CD38^high^) and CD21low B cells (CD19^+^CD21^low^CD38^dim^CD27^−^) were determined. Within the total CD3^+^ T cells, we obtained CD4^+^ and CD8^+^ lineages defined as the following subsets: naive (CD45RO^−^CCR7^+^), central memory T cells (Tcm; CD4RO^+^CCR7^+^), effector memory RO‐positive T cells (TemRO; CD45RO^+^CCR7^−^) and effector memory RA‐positive cells (TemRA; CD45RO^−^CCR7^−^). We determined the naive (CD45RA^+^) and memory (CD45RA^−^) subsets of CD4^+^CD127^−^CD25^+^ regulatory T (Treg) cells. After we excluded the Treg cells, we determined T helper (Th) cell subsets within CD4^+^CD45RA^−^ cells: Th1 CCR6^−^CXCR3^+^CCR4^−^, Th2 CCR6^−^CXCR3^−^CCR4^+^ and Th17 CCR6^+^CXCR3^−^CCR4^+^. We analyzed the data with FacsDIVA software v8 (BD Biosciences), Infinicyt software (Cytognos, Salamanca, Spain) and FlowJo software v10 (FlowJo LLC, Ashland, Oregon). Cell numbers were presented per μL in blood (Looman et al., 2020; van den Heuvel et al., 2017).

### Assessment of attention problem scores

Attention problems were assessed at a median age of 9.8 [25%–75% range 9.6; 9.9] by means of parent‐rated CBCL/6‐18, a questionnaire aimed for children between 6 and 18 years. The CBCL/6‐18 is a well‐validated standard measure of behavioral problems in children (Achenbach & Ruffle, 2000; Hu et al., 2019; Mian et al., 2019). The questionnaire consists of 99 behavioral problem items that can be rated on a three‐point scale: 0 (not true), 1 (somewhat or sometimes true), 2 (very or often true). We used the CBCL attention score that is widely used in epidemiological studies (Achenbach & Ruffle, 2000; Hu et al., 2019; Mian et al., 2019). The following 10 times are included in the CBCL attention problem scale: “acts too young for his/her age,” “fails to finish things,” “cannot concentrate and cannot pay attention for long,” “cannot sit still, restless or hyperactive,” “confused or seems to be in a daze,” “daydreams or gets lost in his/her thoughts,” “impulsive or acts without thinking,” “poor schoolwork,” “inattentive or easily distracted,” “stares blankly.” The internal consistency of the CBCL attention score in this study is 0.80.

### Covariates

We obtained information on child's sex, gestational age and birthweight from midwife and hospital registries (Ask et al., 2018). Child ethnicity was determined based on birth country of parents and categorized as Western and non‐Western, as previously described (Voortman et al., 2015). Child's length and weight were determined at our research center at 10 years and BMI was calculated. Information on maternal age at enrollment, pre‐pregnancy body mass index, smoking during pregnancy, and highest maternal education was derived from parental questionnaires (Hu et al., 2019; Mian et al., 2019; van den Heuvel et al., 2017; Voortman et al., 2015). The Brief Symptom Inventory (BSI) was used to measure maternal psychiatric symptoms during pregnancy at a child's gestational age of 20–25 weeks. The BSI is a self‐reported questionnaire with high validity and reliability to measure the psychological state of an individual in the preceding 7 days (Cents et al., 2013; Modesto et al., 2015; Molenaar et al., 2019). The total score of the BSI was calculated to create the General Symptom Index (GSI), a subscale used to measure maternal psychological symptoms during pregnancy. A total problem scale was created including all 99 items from the CBCL‐6/18, with the exception of the 10 items measuring attention problems to assess other co‐occurring mental health problems.

### Statistical analyses

Because of the presence of the value zero in the attention scores and to approximate a normal distribution of the residuals of our models, we used a LN+1 transformation on the attention scores (outcome) (Figure S1). Cell numbers (determinant) were converted to z‐scores for easier interpretation. Multivariable linear regression analyses were used to determine associations between individual immune cell numbers and attention problem scores. Confounder selection was based on previous studies (van den Heuvel et al., 2017). Confounders were added to the regression model if addition of the confounder resulted in at least 10% change in the beta of the independent variable (Mickey and Greenland, 1989; VanderWeele and Shpitser, 2013). The following covariates were added to the model: child's sex, birth weight, gestational age, child's BMI at 10 years, ethnicity and maternal age at enrollment, highest maternal education, maternal pre‐pregnancy BMI and maternal smoking during pregnancy. To establish whether identified associations are independent of co‐occurring child neurodevelopmental problems we adjusted our models for this total score. Next, we tested possible effect modification by sex by adding an interaction term of sex with cell numbers (Modesto et al., 2015; Murray et al., 2019; Uekert et al., 2006). The percentage of missing data in the covariates ranged from 0.0% to 4.1% with exception for maternal smoking during pregnancy (12.7%), GSI score during pregnancy (15.6%) and maternal pre‐pregnancy BMI (23.1%). We performed multiple imputation by chained equations (*n* = 20) to minimalize potential bias due to missing information on covariates (R package “Mice version 3.6.0”). Determinants and outcome were not imputed. A wide range of predictors, including variables that were not included as confounders, were used as is generally recommended in case of multiple imputation procedures (Collins et al., 2001). To check the process of multiple imputation, characteristics of the imputed and non‐imputed dataset were compared and univariate analyses, multivariate analyses before and after imputation were compared. Because of the natural log transformation for our outcome, pooled regression coefficients were back transformed and can be interpreted as % increase or decrease in attention problem score per 1SD increase in cell number/uL blood. Non‐linearity of the observed associations was considered and tested by adding splines but was not present. No adjustment for multiple testing was performed because of strong correlations between immune cell numbers, leading to dependent tests (Figure S2) (Bender and Lange, 2001; Rothman, 1990). Correlations between covariates are presented in Figure S2. A two‐tailed *p*‐value of <0.05 was considered significant. Statistical analyses were performed using SPSS version 25.0 for Windows (IBM Corp., Armonk, NY, USA) and R version 3.6.1.

## RESULTS

### Subject characteristics

Characteristics of the study population are presented in Table 1. Of the 756 children included, 404 were girls. Highest maternal education was classified as higher in 62.5%, as secondary in 34.5% and as primary in 3.0%. Median GSI during pregnancy was 6.0 (25%–75% range 3.0; 12.0). Median total CBCL problem score was 9.0 (25%–75% range 4.0; 18.7). Characteristics of the study population were comparable in the imputed dataset and overall effect estimates were comparable before and after multiple imputation (Tables S1 and S3). Non‐responder analyses showed that included children had a higher socio‐economic status, were more often Western, had higher birth weight, had less often mothers that smoked and had mothers with a lower gsi (Table S2). Median cell numbers are presented in Table 2.

**TABLE 1:** Characteristics of the study population

Child characteristics (*n* = 756)	General group	Missing in study population (%)
Sex (*N*, %)		0.0
Boy	352 (46.6)	
Girl	404 (53.4)	
Gestational age, wk	40.1 [39.3; 41.0]	0.7
Birth weight, g	3510.0 [3155.0; 3827.5]	0.1
BMI, kg/m^2^	17.0 [15.7; 18.4]	0.0
Ethnicity (*N*, %)		1.3
Western	649 (87.0)	
Non‐western	97 (13.0)	
Attention score CBCL	2.0 [1.0; 5.0]	0.0

**Maternal characteristics**		
Education (*N*, %)		4.1
Primary	22 (3.0)	
Secondary	251 (34.5)	
Higher	455 (62.5)	
Pre‐pregnancy BMI, kg/m^2^	22.6 [20.8; 24.9]	23.1
GSI during pregnancy	0.12 [0.06; 0.2]	15.6
Smoking during pregnancy		12.7
Never	511 (77.4)	
Until pregnancy was known	74 (11.2)	
Continued during pregnancy	75 (11.4)	

*Note*: Table 1 represents the child and maternal characteristics for the study population in the non‐imputed dataset and are represented as number (%) or median [25%–75% range].

Abbreviations: BMI, body mass index; CBCL, Child Behavior Checklist; g, grams; GSI, global severity index; *N*, number; wk, weeks.

**TABLE 2:** Median cell numbers in the study population

T cells	Median cell number per microliter blood (interquartile range)
CD4^+^	
CD4^+^total	1077.8 [880.9; 1288.4]
CD4^+^naive	679.2 [533.6; 869.8]
CD4^+^Tcm	168.6 [110.8; 234.9]
CD4^+^TemRA	131.0 [87.1; 202.9]
CD4^+^TemRO	16.9 [7.8; 37.0]
Th1	31.0 [18.8; 46.3]
Th2	54.7 [38.3; 79.5]
Th17	30.9 [19.8; 43.2]
Th17.1	11.9 [5.6; 20.0]
Treg	78.0 [59.8; 98.1]
Treg naive	45.6 [32.7; 61.9]
Treg memory	29.4 [22.5; 38.7]
CD8^+^	
CD8^+^total	594.1 [483.4; 747.5]
CD8^+^naive	319.0 [240.7; 438.8]
CD8^+^Tcm	22.9 [11.5; 37.5]
CD8^+^TemRA	85.1 [49.6; 138.5]
CD8^+^TemRO	79.2 [50.7; 115.7]
B cells	
B total	482.0 [374.0; 625.9]
B naive	304.8 [231.9; 416.2]
IgG^+^CD27^‐^	4.3 [2.4; 7.2]
IgG^+^CD27^+^	15.7 [9.8; 23.4]
IgA^+^CD27^‐^	2.0 [1.2; 3.2]
IgA^+^CD27^+^	11.3 [7.9; 15.9]
Transitional	49.1 [31.0; 70.5]
CD21^low^	9.7 [6.4; 14.1]

Abbreviations: Ig, immunoglobulin; Tcm, central memory T lymphocytes; TemRA, effector memory RA‐positive T lymphocytes; TemRO, effector memory RO‐positive T lymphocytes; Th, helper T cell; Treg, regulatory T cell.

### Th and Treg cell subsets and attention problems

Children with 1SD higher Th1 cell numbers had 5.3% (95%CI 0.3; 10.5) higher attention problem scores (Table 3). No associations were observed between attention problem score and Treg cell numbers. No associations were observed between either Th2, Th17, naive Treg or memory Treg cells and attention problem scores (Table 3).

**TABLE 3:** Associations between T helper and T regulatory cells and attention problems

	Attention problem score CBCL		
T cells	*β*	95%CI	*p* value
Th1	5.28	(0.33; 10.49)	0.037
Th2	4.49	(−0.55; 9.77)	0.08
Th17	−0.049	(−5.21; 4.47)	0.84
Th17.1	2.14	(−2.17; 7.24)	0.39
Treg	3.98	(−0.96; 9.16)	0.12
Treg naive	2.75	(−2.15; 7.89)	0.28
Treg memory	4.36	(−0.67; 9.65)	0.09
B cells			
B total	0.68	(−3.98; 5.57)	0.78
B naive	1.67	(−3.09; 6.66)	0.50
IgG^+^CD27^‐^	−1.17	(−5.89; 3.78)	0.64
IgG^+^CD27^+^	−2.32	(−6.88; 2.47)	0.34
IgA^+^CD27^‐^	−1.68	(−6.37; 3.25)	0.50
IgA^+^CD27^+^	−1.86	(−6.43; 2.93)	0.44
Transitional	−0.63	(−5.23; 4.18)	0.79
CD21^low^	−1.83	(−6.47; 3.04)	0.45

*Note*: Betas (βs) represent % attention problem score increase or decrease per 1SD increase in cell number/uL blood. Associations are based on multivariable linear regression analyses performed within the imputed dataset and adjusted for child (sex, gestational age, birth weight, BMI, ethnicity, highest maternal education, total problem score) and maternal (pre‐pregnancy BMI, maternal smoking during pregnancy and maternal global severity index during pregnancy) covariates.

Abbreviations: CBCL, Child Behavior Checklist; CI, confidence interval; Ig, immunoglobulin; Th, helper T cell; Treg, regulatory T cell.

### T effector memory cells and attention problems

1SD increase in total CD8^+^ cell numbers was associated with 7.5% (95%CI 2.4; 12.7) higher attention problem scores (Table 4). This increase was mainly explained by an increase in CD8^+^naive and Tcm cell numbers. Namely, 1SD increase in CD8^+^naive cells was associated with 6.9% (95%CI 2.0; 12.1) higher attention problem scores. 1SD increase in CD8^+^Tcm was associated with 6.4% (95%CI 1.5; 11.6) higher attention problem scores. Across CD8^+^ T cells no associations were observed with CD8^+^TemRO or CD8^+^TemRA cell numbers and attention problem scores. Within CD4^+^ T cell numbers, a positive association was observed for CD4^+^Tcm cell numbers and attention problems (5.0% (95%CI 0.2; 10.1)). No associations were observed with other CD4^+^ subset cell numbers (Table 4).

**TABLE 4:** Associations between T effector memory cells and attention problems

	Attention problem score CBCL		
	*β*	95%CI	*p*‐value
CD4^+^			
CD4^+^total	4.34	(−0.60; 9.53)	0.09
CD4^+^naive	2.67	(−2.09; 7.65)	0.28
CD4^+^Tcm	4.99	(0.16; 10.06)	0.04
CD4^+^TemRA	−1.49	(−6.04; 3.28)	0.53
CD4^+^TemRO	0.78	(−4.01; 5.80)	0.76
CD8^+^			
CD8^+^total	7.45	(2.42; 12.72)	0.003
CD8^+^naive	6.90	(1.96; 12.07)	0.006
CD8^+^Tcm	6.41	(1.51; 11.55)	0.01
CD8^+^TemRA	−0.79	(−5.48; 4.15)	0.75
CD8^+^TemRO	1.34	(−3.42; 6.33)	0.59

*Note*: Betas (βs) represent % attention problem score increase or decrease per 1SD increase in cell number/uL blood. Associations are based on multivariable linear regression analyses performed within the imputed dataset and adjusted for child (sex, gestational age, birth weight, BMI, ethnicity, highest maternal education, total problem score) and maternal (pre‐pregnancy BMI, maternal smoking during pregnancy and maternal global severity index during pregnancy) covariates.

Abbreviations: CBCL, Child Behavior Checklist; CI, confidence interval; Tcm, central memory T lymphocytes; TemRA, effector memory RA‐positive T lymphocytes; TemRO, effector memory RO‐positive T lymphocytes.

### B cell subsets and attention problems

No associations were observed between total or naive B cells and attention problems. No associations were observed for CD27^+^ or CD27^‐^ IgG^+^ and IgA^+^ memory B cells, CD21low or transitional B cells and attention problem scores (Table 3).

### Subgroup and sensitivity analyses

Statistical interaction by sex was only observed for CD8^+^TemRO cell numbers. When the analyses were stratified for sex, the association was slightly stronger in boys than in girls. Boys had per 1SD increase in CD8^+^TemRO cell numbers 8.2% (95%CI 1.0; 16.0) higher attention problem scores (Table S4). Results did not change when adjusting for depressive symptoms instead of adjustment for total neurodevelopmental problem score (Table S5).

## DISCUSSION

This population‐based cohort observed that higher numbers of Th1 and CD8^+^, CD8^+^naive and CD8^+^Tcm cells were associated with higher attention problem scores independent of co‐occurring child neurodevelopmental problems. No associations were observed between Th2, Th17 and CD4^+^ effector memory subsets and attention problem scores. No associations were observed between memory B cells and attention problem scores.

Our study contributed to literature by the determination of associations between B and T cell numbers and attention problems with adjustment for important possible confounders. The possibility of an altered adaptive immune cell composition has been suggested previously but not yet been studied (Zhou et al., 2017). The availability of data from a large‐scale population‐based cohort of children was a unique setting to determine this. Previous studies on associations between attention problems and the immune system are highly heterogeneous in study population and study design. However, a systematic review reported associations between immunological inflammation and attention problems (Anand et al., 2017). We here extended previous literature by studying peripheral circulating immune cells in relation to attention problems in children from a population‐based birth cohort study. Because of the high heterogeneity in studies on immunological markers and attention problems, direct comparison of our results to previous literature is not straightforward. However, in accordance with previous evidence pointing towards a pro‐inflammatory mediated immune system, we observed higher Th1 cells in children with higher attention problem scores (Anand et al., 2017; Verlaet et al., 2019). No studies on T and B cells in attention problems have been reported previously. Yet, IL‐1β, IL‐2, IFN‐ɣ and TNF‐α, pro‐inflammatory cytokines produced by Th1 cells as well as other immune cells such as other lymphocytes and macrophages, have been linked to ADHD or ADHD symptoms in previous studies (Anand et al., 2017; Cortese et al., 2019; Leffa et al., 2018). In addition, ADHD genetic risk has been associated with Th1‐mediated diseases such as psoriasis, rheumatoid arthritis and ADHD diagnosis with type 1 diabetes and autoimmune thyroiditis (Hoekstra, 2019; Leffa et al., 2018; Verlaet et al., 2014).

In contrast with previous studies that suggested increased Th2 cells because of the association of attention problems with atopic disease, we did not observe an association between attention problems and Th2 cell numbers (Cortese et al., 2018; Pelsser et al., 2009; Verlaet et al., 2019). Previously, it has been shown that in chronic atopic dermatitis immunological inflammation could cause a shift towards increased Th1 and CD8^+^T cell numbers (Hijnen et al., 2013; Knol and Hijnen, 2016). This might explain the association of attention problems with both Th1 and Th2‐mediated disease as well as our study results with increased Th1, CD8^+^ cells, but not Th2 cells. In addition, another explanation could be the presence of different ADHD phenotypes which has been suggested previously because of inter‐individual differences (Anand et al., 2017; Leffa et al., 2018). Moreover, we here studied attention problems as a continuous spectrum within the general population, as opposed to ADHD in clinical samples. Hence, it is important to test to what extent our findings can be generalized at more extreme ends of symptom severity.

Next to higher Th1 cells, we observed higher total CD8^+^, CD8^+^naive and CD8^+^Tcm cell numbers in children with more attention problems. Tcm cells express CCR7 and L‐selectin, leading to circulation in blood and the primary and secondary lymphoid organs (Sallusto et al., 2004). Despite previous research hypothesizing an association between CD8^+^ cells and attention problems, this is the first study to examine and support such an association (Zhou et al., 2017).

Various underlying mechanisms could be speculated. First, Th1 and CD8^+^ cells could contribute to the pathogenesis of attention problems. In other neuropsychiatric diseases a cross‐talk between the peripheral immune system and cerebral immunity has been shown (Bennett and Molofsky, 2019; Lasselin et al., 2018). CD8^+^ T cells are thought to be involved in neuroinflammation by blood‐brain barrier disruption (Xie & Yang, 2015). However, these neuroinflammatory processes with alterations in immune cell numbers have never been studied in relation to specifically attention problems and require further study. Besides the adaptive immunity, a role for the innate immunity in the pathophysiology of attention problems is possible (Song et al., 2020). Second, it could be speculated that altered neuronal processes as observed in attention problems cause changes in the peripheral immunity. Protocatherins (Pcdhs) are involved in neuronal development and have been implicated in the pathogenesis of ADHD (Morimoto and Nakajima, 2019; Peek et al., 2017). Pcdhs also have immunomodulatory functions and specifically Pcdhs18 is an activator marker of CD8^+^ T cells and inhibitor of the effector CD8^+^ T cells (Morimoto & Nakajima, 2019; Vazquez‐Cintron et al., 2012). This might explain our observation of higher CD8^+^naive and CD8^+^Tcm cells but not effector memory cells. Third, attention problems have been associated with oxidative stress through reactive oxygen species, psychological stress and diet (Alvarez‐Arellano et al., 2020; Mian et al., 2019; Verlaet et al., 2019). Previous studies show that children with attention problems have higher risks for unhealthy diets and obesity (Cortese et al., 2019; Mian et al., 2019). Thus, increased levels of Th1 and CD8^+^ could reflect oxidative stress (Solleiro‐Villavicencio & Rivas‐Arancibia, 2018). More studies are needed to further unravel the possible role of an immunological pathophysiology in attention problems and to determine if there exists a causal relationship between adaptive immunity and attention problems.

Because multiple psychiatric conditions have been associated with inflammation it could be argued that the observed associations are due to other psychiatric conditions. To account for other behavioral or neurodevelopmental problems, we adjusted for concurrent neurodevelopmental problems. Interestingly, the observed associations were independent of total CBCL problem score and depressive symptom score, supporting an association independent of other psychiatric conditions (Mac Giollabhui et al., 2021).

Finally, we observed differences in associations between boys and girls. While the direction of associations observed for total CD8^+^, CD8^+^naive and CD8^+^Tcm with attention problems were consistent across sex, slightly stronger associations were observed in boys, in particular for CD8^+^TemRO. Previous studies on ADHD report sex differences in ADHD symptoms across childhood (Murray et al., 2019; Uekert et al., 2006). Girls tend to have lower scores on hyperactivity, inattention, impulsivity and externalizing problems than boys which could explain the stronger associations in boys (Murray et al., 2019; Uekert et al., 2006).

An important strength of our study is the extensive assessment of immune cell numbers in relation to dimensional attention problems in a population‐based setting. We had the ability to adjust the associations for confounders including other child psychopathologies in contrast to previous studies. However, the following limitations should be taken into consideration. First, no information on clinical diagnosis of ADHD in children was available. This limits the generalizability of our findings to a clinical setting in children with ADHD diagnosis. Instead, we used the well‐validated parental‐reported CBCL questionnaires to measure attention problems dimensionally, as used previously in epidemiological studies (Hu et al., 2019; Katsuki et al., 2020; Mian et al., 2019; Modesto et al., 2015). This method has a higher feasibility than daily ADHD symptom measurements but might be less accurate (Mian et al., 2019). In addition, we were unable to study the subgroup of children on ADHD medication as the number of children with medication was too low. Residual confounding and the possibility of chance finding due to multiple tests cannot be ruled out and the present exploratory findings should be considered as hypothesis‐generating (Rothman, 1990). Non‐responder analyses showed that responders differed in characteristics compared with non‐responders. This might limit the generalizability of the study to other populations such as populations with a lower socio‐economic status. Finally, cause‐effect relationships between immune cells and attention problems cannot be established because of the cross‐sectional design of this study.

## CONCLUSION

Higher Th1, total CD8^+^, CD8^+^naive, CD8^+^Tcm cell numbers are observed in children with more attention problems, independent of other childhood psychopathology. The results suggest that children with attention problems have a skewed balance towards Th1 cells leading to higher cytotoxic T cell numbers.

## CONFLICT OF INTEREST

No conflicts of interest.

## ETHICS STATEMENT

The Medical Ethical Committee of the Erasmus University Medical Center in Rotterdam approved the study (MEC‐2012‐165). Written informed consent from legal representatives was obtained from all participants.

## AUTHOR CONTRIBUTIONS

Kirsten I. M. Looman, Charlotte A. M. Cecil, Jessica C. Kiefte‐de Jong, Menno C. van Zelm, Henriëtte A. Moll contributed to the study design and interpretation of the data. Menno C. van Zelm designed and supervised the flow cytometry experiments. Christina Grosserichter‐Wagener performed the flow cytometry experiments. Kirsten I. M. Looman had full access to all of the data and performed the data analyses. Kirsten I. M. Looman, Charlotte A. M. Cecil, Menno C. van Zelm, Henriëtte A. Moll wrote the manuscript. All authors critically revised the article for important intellectual content and gave final approval of the version to be published. Henriëtte A. Moll supervised the study.

## Supporting information

Supplementary Material 1

Supplementary Material 2

Supplementary Material 3

## Data Availability

The datasets generated during and/or analyzed during the current study are not publicly available but are available from the corresponding author on reasonable request.
