# Supplementary material for: Associations between T cells and attention problems in the general pediatric population: The Generation R study
Source: JCPP Adv. 2021 Oct 13;1(3):e12038. doi: 10.1002/jcv2.12038 (PMC10242894; doi:10.1002/jcv2.12038)
Supplement: Supplementary file 1 — Supplementary Material 1 [file JCV2-1-e12038-s002.docx]

**Supplemental Tables File**

**Associations between T cells and attention problems in the general pediatric population: the Generation R Study.**

**Running head**: T cells and child attention problems

Kirsten I.M. Looman^1,2^, Charlotte A.M. Cecil^3,4,5^, Christina Grosserichter-Wagener^6^,

Jessica C. Kiefte-de Jong^4,7^, Menno C. van Zelm^8^, Henriëtte A. Moll^2^

^1^ Generation R Study Group, Erasmus MC, University Medical Center, Rotterdam, the Netherlands
^2^ Department of Pediatrics, Sophia Children’s Hospital, Erasmus MC, University Medical Center, Rotterdam, the Netherlands

^3^ Department of Child and Adolescent Psychiatry/Psychology, Erasmus MC, University Medical Center, Rotterdam, the Netherlands
^4^ Department of Epidemiology, Erasmus MC, University Medical Center, Rotterdam, the Netherlands

^5^ Molecular Epidemiology, Department of Biomedical Data Sciences, Leiden University Medical Center, Leiden, the Netherlands

^6^ Department of Immunology, Erasmus MC, University Medical Center, Rotterdam, the Netherlands

^7^ Department of Public Health and Primary Care/LUMC Campus The Hague, Leiden University Medical Center, Leiden, The Netherlands ^8^ Department of Immunology and Pathology, Central Clinical School, Monash University and Alfred Hospital, Melbourne, Victoria, Australia

**SUPPLEMENTAL TABLES (n=5)**

**Table S1**. Characteristics of the study population in the imputed dataset

| **Child characteristics (n=756)** | **General group** |
| --- | --- |
| Sex (N, %) |  |
| Boy | 353 (46.6) |
| Girl | 404 (53.4) |
| Gestational age, wk | 40.1 [39.3;41.0] |
| Birth weight, g | 3510.0 [3150.0; 3826.3] |
| BMI, kg/m^2^ | 17.0 [15.8; 18.4] |
| Ethnicity (N, %) |  |
| Western | 655 (86.6) |
| Non-western | 101(13.4) |
| Attention score CBCL | 2.0 [1.0;5.0] |
| **Maternal characteristics** |  |
| Education (N, %) |  |
| Primary | 25 (3.3) |
| Secondary | 264 (34.9) |
| Higher | 467 (61.8) |
| Pre-pregnancy BMI, kg/m^2^ | 22.7 [20.8; 25.0] |
| GSI during pregnancy | 0.1 [0.06;0.23] |
| Smoking during pregnancy |  |
| Never | 580 (76.7) |
| Until pregnancy was  known | 92 (12.2) |
| Continued during  pregnancy | 84 (11.1) |
| Abbreviations: BMI, body mass index; CBCL, Child Behavior Checklist; g, grams; GSI, global severity index; N, number; wk, weeks.  Table S1 represents the child and maternal characteristics for the study population. Values are based on the imputed dataset (analyzed sample) and represented as number (%) or median [25-75% range]. | |

**Table S2. Non responder-analyses**

| **Child characteristics** | **Responders (n=756)** | **Non-responders**  **(n=5106)** |
| --- | --- | --- |
| Sex (N, %) |  |  |
| Boy | 352 (46.6) | 2561 (50.2) |
| Girl | 404 (53.4) | 2544 (49.8) |
| Gestational age, wk | 40.1 [39.3; 41.0] | 40.1 [39.0; 41.0] |
| Birth weight, g | 3510.0 [3155.0; 3827.5] | 3420.0 [3050;3768.8] |
| BMI, kg/m^2^ | 17.0 [15.7;18.4] | 16.9 [15.7;18.8] |
| Ethnicity (N, %) |  |  |
| Western | 649 (87.0) | 3267 (65.8) |
| Non-western | 97 (13.0) | 1700 (34.2) |
| Attention score CBCL | 2.0 [1.0;5.0] | 2.0 [1.0;5.0] |
| **Maternal characteristics** |  |  |
| Education (N, %) |  |  |
| Primary | 22 (3.0) | 386 (8.3) |
| Secondary | 251 (34.5) | 2008 (34.2) |
| Higher | 455 (62.5) | 2251 (48.5) |
| Pre-pregnancy BMI, kg/m^2^ | 22.6 [20.8; 24.9] | 22.6 [20.8;25.3] |
| GSI during pregnancy | 0.12 [0.06;0.2] | 0.15 [0.08;0.35] |
| Smoking during pregnancy |  |  |
| Never | 511 (77.4) | 3391 (76.2) |
| Until pregnancy was  known | 74 (11.2) | 376 (8.5) |
| Continued during  pregnancy | 75 (11.4) | 681 (15.3) |
| Abbreviations: BMI, body mass index; CBCL, Child Behavior Checklist; g, grams; GSI, global severity index; N, number; wk, weeks. | | |

Table S2 compares the characteristics of the study population (n=756) versus the children that visited the research center but were not included in the current study because of no data on the immune cell numbers or attention problem score at 10 years of age. Values are based on the non-imputed dataset and represented as number (%) or median [25-75% range].

**Table S3. Analyses before multiple imputation**

|  | **Attention score CBCL** | |
| --- | --- | --- |
| **CD4^+^** | **β** | **95%CI** |
| CD4^+^total | 5.2 | (-1.0;11.9) |
| CD4^+^naive | 4.4 | (-1.6; 10.7) |
| CD4^+^Tcm | 4.3 | (-1.6; 10.6) |
| CD4^+^TemRO | -0.3 | (-6.4; 6.1) |
| CD4^+^TemRA | -0.7 | (-5.9; 4.9) |
| Th1 | 10.9 | (4.5; 17.7) |
| Th2 | 1.3 | (-4.5; 7.6) |
| Th17 | -4.9 | (-10.7; 1.3) |
| Th17.1 | 4.8 | (-1.2; 11.2) |
| Treg | 3.4 | (-2.7; 9.9) |
| Treg naive | 2.4 | (-3.5; 8.6) |
| Treg memory | 3.9 | (-2.5; 10.7) |
| **CD8^+^** |  |  |
| CD8^+^total | 4.4 | (-1.3; 11.3) |
| CD8^+^naive | 8.0 | (1.6; 14.8) |
| CD8^+^Tcm | 4.8 | (-1.5; 11.5) |
| CD8^+^TemRO | -0.5 | (-6.5; 6.0) |
| CD8^+^TemRA | 0.7 | (-5.3; 6.1) |
| **B cells** |  |  |
| B total | 0.8 | (-5.0; 6.7) |
| B naive | 1.9 | (-4.0; 8.2) |
| IgG^+^ CD27^-^ | -2.7 | (-8.3; 3.3) |
| IgG^+^ CD27^+^ | -3.5 | (-9.1; 2.6) |
| IgA^+^ CD27^-^ | -2.6 | (-8.2; 3.4) |
| IgA^+^ CD27^+^ | -1.8 | (-7.5; 4.3) |
| Transitional | -2.3 | (-7.6; 3.3) |
| CD21^low^ | -0.3 | (-5.9; 5.8) |
| Abbreviations: CI, confidence interval; CBCL, Child Behavior Checklist; Tcm, central memory T lymphocytes; TemRA, effector memory RA-positive T lymphocytes; TemRO, effector memory RO-positive T lymphocytes; Th, helper T cell; Treg, regulatory T cell, Ig, immunoglobulin.  Betas (βs) represent *%* attention problem score increase or decrease per 1SD increase in cell number/uL blood. The associations are based on multivariable linear regression analyses and adjusted for child (sex, gestational age, birth weight, BMI, ethnicity, highest maternal education, total problem score) and maternal (pre-pregnancy BMI, maternal smoking during pregnancy and maternal global severity index during pregnancy) covariates. Number of children included is 486 to 505 dependent on the individual because of the exclusion of children with any missing on any covariate. | | |

**Table S4.** Associations between T cells and attention problem scores stratified by child’s sex

|  | **Boys (N=352)** | | | **Girls (N=404)** | | |
| --- | --- | --- | --- | --- | --- | --- |
|  | **β** | **95%CI** | **P value** | **β** | **95%CI** | **P value** |
| Th1 | 10.86^a^ | (2.27;20.17 | 0.01 | 3.64 ^g^ | (-2.61; 10.29) | 0.26 |
| Th2 | 6.89^a^ | (-1.56;15.63) | 0.11 | 2.86 ^h^ | (-3.48; 9.62) | 0.39 |
| Th17 | -0.36 ^b^ | (-8.21; 8.16) | 0.93 | -0.60 ^i^ | (-6.52; .570) | 0.85 |
| Th17.1 | 3.67 ^b^ | (-3.98;11.93) | 0.36 | 1.96 ^i^ | (-4.38; 8.74) | 0.55 |
| Treg | 6.83 ^c^ | (-0.54; 14.75) | 0.07 | 1.80 ^i^ | (-4.80;8.85) | 0.60 |
| Treg naive | 4.70 ^d^ | (-2.51; 12.43) | 0.21 | 1.06 ^k^ | (-5.57; 8.16) | 0.76 |
| Treg memory | 6.84 ^d^ | (-0.57 14.80) | 0.07 | 2.45 ^k^ | (-4.36; 9.73) | 0.49 |
| **CD8^+^** |  |  |  |  |  |  |
| CD8**^+^** total | 12.86 ^e^ | (5.38; 20.87) | 0.001 | 3.84 ^l^ | (-2.99; 11.15) | 0.28 |
| CD8**^+^** naive | 8.24 ^f^ | (1.18;15.80) | 0.02 | 6.14 ^m^ | (-0.81; 13.57) | 0.09 |
| CD8**^+^**Tcm | 10.09  ^f^ | (2.58; 18.14) | 0.008 | 4.73 ^m^ | (-1.84; 11.74) | 0.16 |
| CD8**^+^**TemRA | 2.98  ^f^ | (-3.28; 9.65) | 0.36 | -5.27 ^m^ | (-12.22; 2.23) | 0.16 |
| CD8**^+^**TemRO | 8.22 ^f^ | (0.96; 16.01) | 0.03 | -3.27 ^m^ | (-9.59; 3.49) | 0.34 |
| Abbreviations: CI, confidence interval; Tcm, central memory T lymphocytes; TemRA, effector memory RA-positive T-lymphocytes; TemRO, effector memory RO-positive T-lymphocytes.  Betas (βs) represent % attention problem score increase or decrease per 1SD increase in cell number/uL blood. The associations are based on multivariable linear regression analyses and adjusted for child (gestational age, birth weight, BMI, ethnicity, highest maternal education, total problem score) and maternal (pre-pregnancy BMI, maternal smoking during pregnancy and maternal global severity index during pregnancy) covariates.  ^a n=335, b n=334, c n=330, d n=329, e n=333, f n=343^  ^g n=383, h n=382, i n= 379, k n=377, l n=385, m n=396^ | | | | | | |

**Table S5. Analyses adjusted for depression instead of total CBCL**

|  | **Attention score CBCL** | |
| --- | --- | --- |
| **CD4^+^** | **β** | **95%CI** |
| CD4^+^total | 3.61 | (-1.87;9.39),p=0.20 |
| CD4^+^naive | 1.65 | (-3.60;7.18),p=0.55 |
| CD4^+^Tcm | 4.77 | (-0.61;10.43),p=0.08 |
| CD4^+^TemRA | -1.23 | (-6.31;4.13),p=0.65 |
| CD4^+^TemRO | 1.73 | (-3.65;7.42),p=0.54 |
| Th1 | 5.67 | (0.10;11.56),p=0.046 |
| Th2 | 4.45 | (-1.12;10.34),p=0.12 |
| Th17 | -1.43 | (-6.66;4.11),p=0.61 |
| Th17.1 | 2.74 | (-2.71;8.50),p=0.33 |
| Treg | 4.37 | (-1.13;10.18), p=0.12 |
| Treg naive | 2.75 | (-2.69;8.49), p=0.33 |
| Treg memory | 5.01 | (-0.61;10.94), p=0.08 |
| **CD8^+^** |  |  |
| CD8^+^total | 7.59 | (1.96;13.51),p=0.008 |
| CD8^+^naive | 6.31 | (0.83;12.09),p=0.02 |
| CD8^+^Tcm | 6.52 | (1.04;12.29),p=0.02 |
| CD8^+^TemRA | -1.31 | (-6.52;4.19),p=0.63 |
| CD8^+^TemRO | 2.67 | (-2.70;8.34),p=0.34 |
| **B cells** |  |  |
| B total | 0.12 | (-5.06;5.58),p=0.97 |
| B naive | 1.27 | (-4.03;6.86),p=0.65 |
| IgG^+^CD27^-^ | -0.25 | (-5.58;5.38),p=0.93 |
| IgG^+^CD27^+^ | -2.22 | (-7.26;3.22),p=0.43 |
| IgA^+^CD27^-^ | -0.98 | (-6.26;4.61),p=0.73 |
| IgA^+^CD27^+^ | -1.99 | (-7.09;3.38),p=0.46 |
| Transitional | 1.42 | (-6.52;3.95),p=0.60 |
| CD21^low^ | -2.93 | (-8.04;2.46),p=0.28 |
| Abbreviations: CI, confidence interval; CBCL, Child Behavior Checklist; Tcm, central memory T lymphocytes; TemRA, effector memory RA-positive T lymphocytes; TemRO, effector memory RO-positive T lymphocytes; Th, helper T cell; Treg, regulatory T cell, Ig, immunoglobulin.  Betas (βs) represent *%* attention problem score increase or decrease per 1SD increase in cell number/uL blood. The associations are based on multivariable linear regression analyses and adjusted for child (sex, gestational age, birth weight, BMI, ethnicity, depression problem score) and maternal (pre-pregnancy BMI, maternal smoking during pregnancy, maternal global severity index during pregnancy, highest maternal education) covariates.  The following questions are included in the 13 item depressive/anxious symptom CBCL score: fears he/she might think or do something bad, he/she has to be perfect, feels or complains no one loves him/her, feels worthless or inferior, nervous/highstrung/tense, too fearful or anxious, feels too guilty, self-conscious or easily embarrassed, talks about killing self, worries, fears he/she might think or do something bad, fears going to school, fears certain animals/ situations/ or places other than school (Cronbach’s alpha=0.77). | | |
