# Supplementary material for: Associations between T cells and attention problems in the general pediatric population: The Generation R study
Source: JCPP Adv. 2021 Oct 13;1(3):e12038. doi: 10.1002/jcv2.12038 (PMC10242894; doi:10.1002/jcv2.12038)
Supplement: Supplementary file 2 — Supplementary Material 2 [file JCV2-1-e12038-s003.pdf]

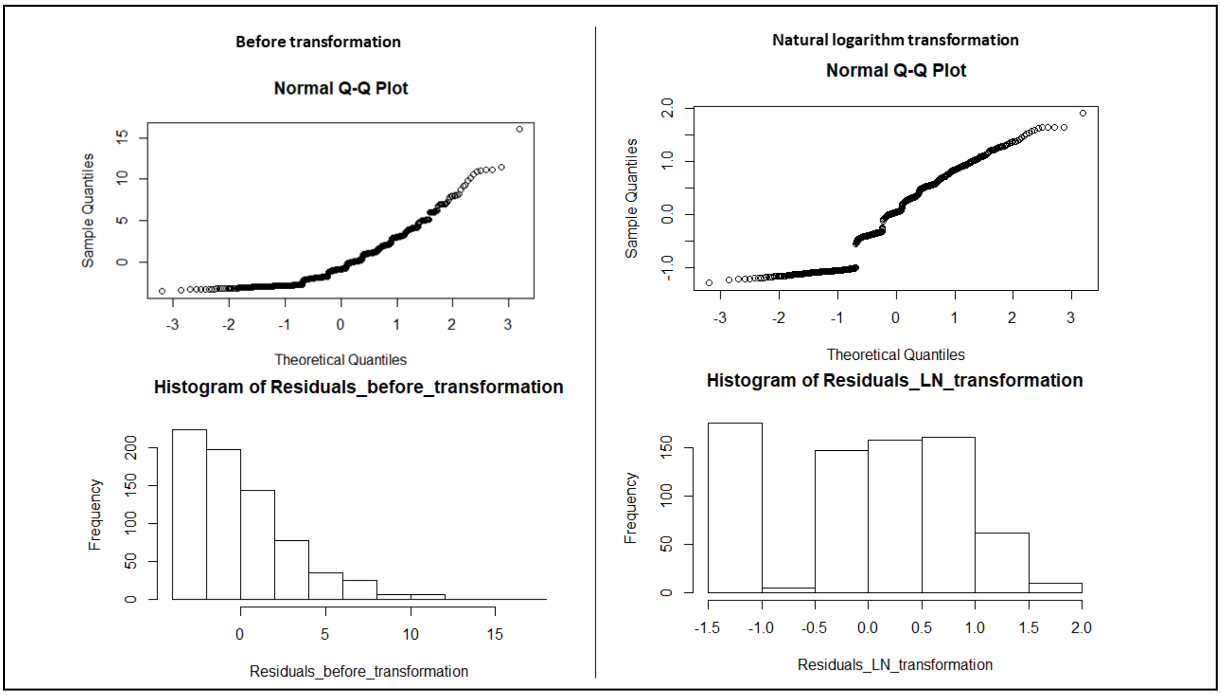

Figure S1. Information on natural logarithm transformation and model fit. Figure S1 shows the model in which Th1 is used as determinant. The model before transformation has a skewness of 1.34 and a kurtosis of 5.3. The model after natural logarithm transformation has a skewness of -0.04 and a kurtosis of 1.9, corresponding to a platykurt model.
