# Supplementary material for: Associations between T cells and attention problems in the general pediatric population: The Generation R study
Source: JCPP Adv. 2021 Oct 13;1(3):e12038. doi: 10.1002/jcv2.12038 (PMC10242894; doi:10.1002/jcv2.12038)
Supplement: Supplementary file 3 — Supplementary Material 3 [file JCV2-1-e12038-s001.pdf]

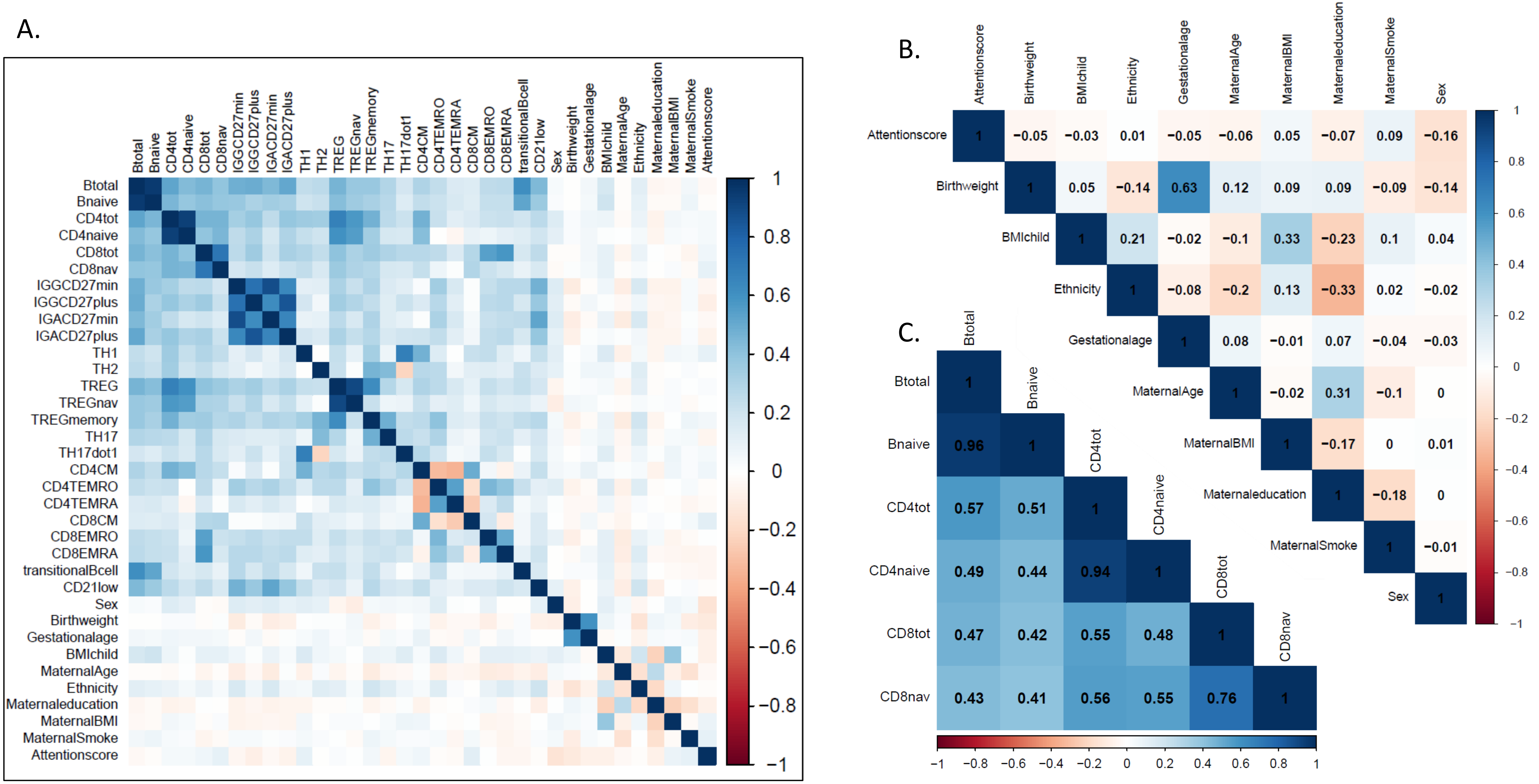
A.
Btotal
Bnaive
CD4tot
CD4naive
CD8tot
CD8nav
IGGCD27min
IGGCD27plus
IGACD27min
IGACD27plus
TH1
TH2
TREG
TREGnav
TREGmemory
TH17
TH17dot1
CD4CM
CD4TEMRO
CD4TEMRA
CD8CM
CD8EMRO
CD8EMRA
transitionalBcell
CD21low
Sex
Birthweight
Gestationalage
BMIchild
MaternalAge
Ethnicity
Maternaleducation
MaternalBMI
MaternalSmoke
Attentionscore
1
0.8
0.6
0.4
0.2
0
−0.2
−0.4
−0.6
−0.8
−1
B.
Attentionscore
Birthweight
BMIchild
Ethnicity
Gestationalage
MaternalAge
MaternalBMI
Maternaleducation
MaternalSmoke
Sex
Attentionscore 1 −0.05 −0.03 0.01 −0.05 −0.06 0.05 −0.07 0.09 −0.16
Birthweight 1 0.05 −0.14 0.63 0.12 0.09 0.09 −0.09 −0.14
BMIchild 1 0.21 −0.02 −0.1 0.33 −0.23 0.1 0.04
Ethnicity 1 −0.08 −0.2 0.13 −0.33 0.02 −0.02
Gestationalage 1 0.08 −0.01 0.07 −0.04 −0.03
MaternalAge 1 −0.02 0.31 −0.1 0
MaternalBMI 1 −0.17 0 0.01
Maternaleducation 1 −0.18 0
MaternalSmoke 1 −0.01
Sex 1
1
0.8
0.6
0.4
0.2
0
−0.2
−0.4
−0.6
−0.8
−1
C.
Btotal
Bnaive
CD4tot
CD4naive
CD8tot
CD8nav
Btotal 1
Bnaive 0.96 1
CD4tot 0.57 0.51 1
CD4naive 0.49 0.44 0.94 1
CD8tot 0.47 0.42 0.55 0.48 1
CD8nav 0.43 0.41 0.56 0.55 0.76 1
−1 −0.8 −0.6 −0.4 −0.2 0 0.2 0.4 0.6 0.8 1

D.

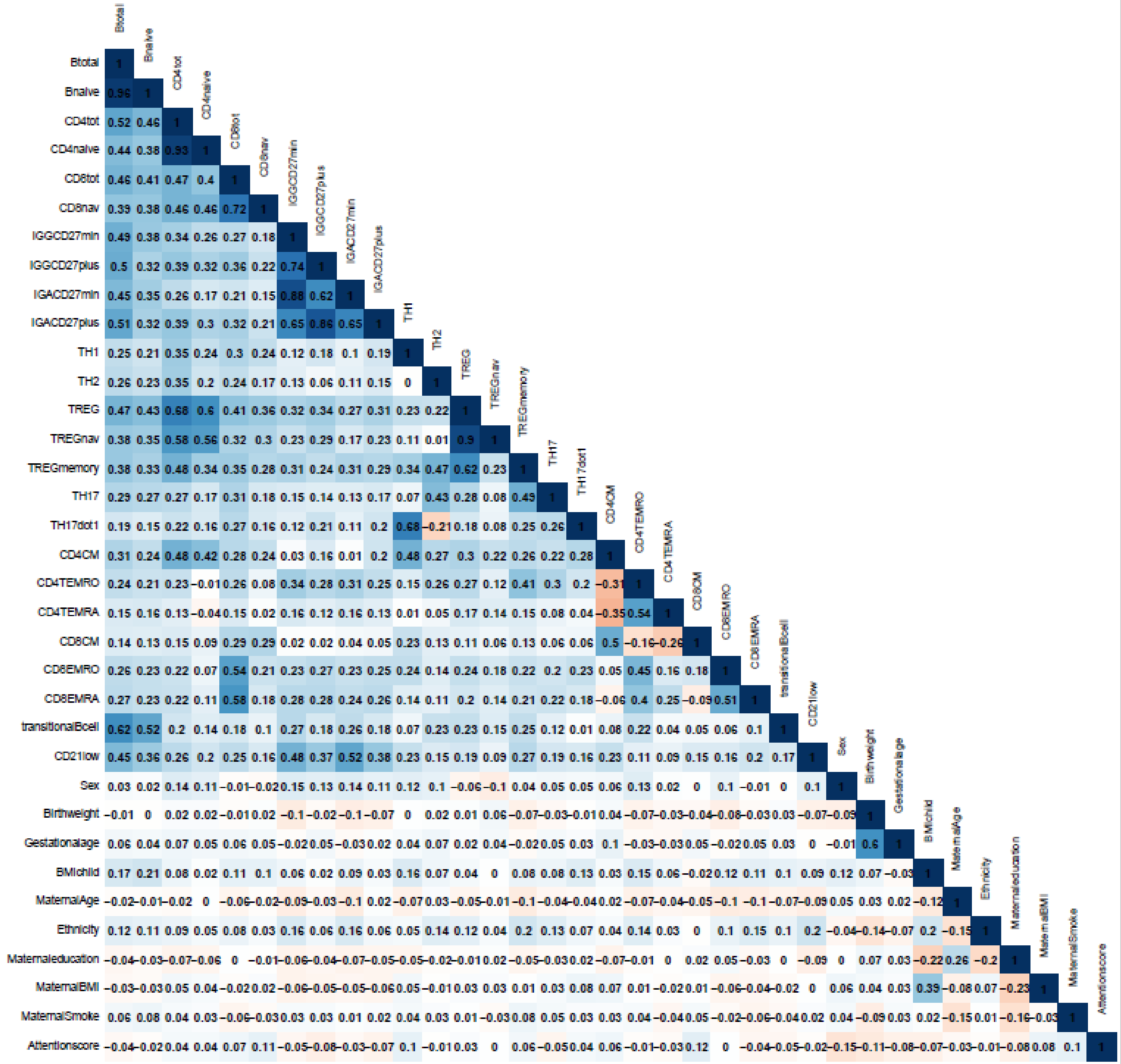

Supplemental Figure 2. Correlation Plot of dependent and independent variables
Figure S2 shows the Pearson correlation coefficients between

A. all included variables and **B.** confounding factors and attention problem score **C.** main B and T cell lineages and **D.** all included variables in detail
